# Professor Busch

**Published:** 1858-09

**Authors:** 


PROFESSOR BUSCH.—In our last issue we announced the death of the distinguished anatomist and physiologist, Prof. Müller, and we have now to record the decease of Deitrich Wilhelm Heinrich Busch, Professor of Midwifery in the University of Berlin, which has thus, in the space of a few months, lost two of its most renowned professors. He was apparently recovering from an attack of acute rheumatism, when cardiac disease supervened, and he died suddenly in the seventieth year of his age. He was the author of several works in his particular branch, which were well appreciated by his readers. Early in life he entered the army, and was, in 1814, made Professor of Surgery at Marburg. He soon gave up this branch for that of Midwifery, and was appointed, in 1827, to the chair which he so ably filled up to the day of his death.

